# Effects of microbial agent application on the bacterial community in ginger rhizosphere soil under different planting years

**DOI:** 10.3389/fmicb.2023.1203796

**Published:** 2023-09-07

**Authors:** Qian Wang, Juan Song, Jinlian Zhang, Xiaojuan Qin, Yihao Kang, Shilv Huang, Shengmao Zhou, Tingsu Chen

**Affiliations:** ^1^Microbiology Research Institute, Guangxi Academy of Agricultural Sciences, Nanning, China; ^2^Vegetable Research Institute, Guangxi Academy of Agricultural Sciences, Nanning, China

**Keywords:** ginger, ginger wilt disease, microbial agents, rhizosphere, bacterial community

## Abstract

Ginger is one of the important spice crops in the world. Due to the prevalence of ginger wilt disease and the lack of effective prevention and control methods, the planting area, total production and value have declined sharply, which have become a key factor restricting ginger industry development in China. Understanding the influence of microbial agents on the rhizosphere microbiota of ginger will facilitate developing novel technologies for the prevention and control of ginger wilt disease. In the new planting and continuous cropping ginger fields, using large-root ginger and microbial agents, two inoculation levels (inoculation and no inoculation) were designed, and high-throughput sequencing technology was used to study the bacterial community structure in the rhizosphere soil at mature stage of ginger. The results showed that newly planted ginger showed a significant yield advantage over continuous cropping ginger, with a yield increase of 39% to 56%, and the lowest ginger wilt disease index. The community structure at the phylum level of soil bacteria in each treatment was very similar to that in the control, but the abundance of some taxonomic units changed significantly. The four dominant phyla of bacteria in mature ginger rhizosphere soil were *Proteobacteria*, *Actinobacteria*, *Chloroflexi*, and *Acidobacteria*, accounting for 72.91% to 89.09% of the total. The microbial agent treatment of continuous cropping had beneficial microorganisms such as *Acidobacteria* and *Gemmatimonadetes* with abundances increased by 12.2% and 17.1%, respectively, compared to the control. The microbial inoculant treatment of newly planted ginger increased the abundance of *Acidobacteria* and *Gemmatimonadetes* by 34.4% and 10.7%, respectively, compared to the control. The composition of bacterial communities were affected by changes in soil properties. Redundancy analysis showed that the hydrolysable nitrogen, available phosphorus, available potassium, and organic matter were significantly related to the composition of soil bacterial communities. Therefore, the microbial agents can not only promote the proliferation of beneficial microorganisms in the continuous cropping soil but also further reshape the soil bacterial community structure by changing the soil physicochemical properties such as effective phosphorus. These results provided a reference for related research on the impact of ginger continuous cropping on soil environment and soil management improvement in ginger fields.

## 1. Introduction

Ginger (*Zingiber officinale* Rosc.) is one of the most important spice crops in China, as well as in several other countries including India, Nepal, Indonesia, and Nigeria (Thekkan et al., 2020). Ginger is widely grown in China and has gradually developed into a large-scale and industrialized production, becoming an important source of income for farmers. However, ginger has a serious problem of continuous cropping obstacles, which leads to significant yield decrease and economic loss. The continuous cropping obstacle for ginger is mainly caused by ginger wilt disease caused by *Ralstonia solanacarum*, which does not occur when the soil and ginger seeds do not carry the pathogen. The incidence rate of ginger wilt disease is 60% when only the soil carries the pathogen, while it is 90% when both the soil and ginger seeds carry the pathogen (Wang et al., 2016). Currently, the methods for the prevention and control of ginger wilt disease mainly include strengthening field management, cultivating resistant varieties, using chemical agents, and applying biocontrol agents (Ohike et al., 2013; Thomas and Upreti, 2014). However, it has been shown that field management is difficult to effectively control the occurrence of ginger wilt disease, the breeding of resistant plants is time-consuming, and chemical control pollutes the environment, disrupts the ecological balance, and long-term use of chemical agents can lead to the pathogen developing resistance. Use of safe biological products is currently a trend (Guo et al., 2004). Therefore, use of beneficial microorganisms to replace harmful chemicals has aroused great interest (Kwon and Kim, 2014).

Microbial agents are microbial live preparations with a certain porous substance as a carrier, which can reproduce in soil or substrate and form an advantageous microbial community for plant growth (Hou et al., 2016). They can improve the soil microbial flora and metabolic activity, prevent and control soil-borne diseases, and thus have a significant promoting effect on plant growth. However, considering that *R. solanacarum* is a highly destructive soil-borne bacterium that can survive for a long time in soil, water, and plants, a single microbial agent has poor efficacy and is subject to problems such as poor persistence and strong environmental dependence, making it difficult to achieve ideal control effect (Huang et al., 2013). In contrast, use of composite microbial inoculant for biological control has become a new research hotspot, and the focus is the compound of beneficial bacteria. Reports on use of composite microbial agents to control *R. solanacarum* mainly focus on the combination of beneficial bacterial strains. For example, Wang et al. (2017) mixed two strains of *Bacillus* isolated from the healthy tomato rhizosphere, and the effect on controlling tomato bacterial wilt was significantly better than that of a single strain. Huang et al. (2015) mixed *Bacillus amyloliquefaciens* and *Streptomyces sulfate* to control tobacco bacterial wilt, achieving an efficacy of 65.85% and reducing the amount of chemical pesticides used by half. Therefore, it is important to improve the control effect of *R. solanacarum* on ginger by using composite microbial agents.

Arbuscular mycorrhiza (AM) fungi, *Trichoderma* spp., and plant growth-promoting rhizobacteria (PGPR), widely distributed, are important soil beneficial bacteria (Afaque et al., 2017; Che et al., 2019). AM fungi can form mutualistic associations with the roots of most terrestrial plants and promote the absorption and utilization of water and mineral elements, particularly nitrogen, phosphorus, and potassium, benefiting plant growth and development (Smith and Read, 2008; Sharifi, et al., 2007). PGPR includes a variety of soil microorganisms, such as nitrogen-fixing bacteria *Azotobacter* sp., and certain species of *Pseudomonas* and *Bacillus* sp. (Glick et al., 1998). *Trichoderma* spp. is a common type of fungus in soil. Studies have shown that *Trichoderma* has biocontrol effects, has the ability to dissolve insoluble inorganic phosphates and potassium in soil, and can promote the germination of various plant seeds and the growth of seedlings (Zhang et al., 2012). To date, many studies have reported the disease control effects of *Trichoderma* against tomato bacterial wilt and tobacco bacterial wilt (Zhu and Yao, 2014; Tan et al., 2011), but its effect on ginger disease control and the root soil microbial community has not received enough attention.

Rhizosphere microorganisms are important components of soil ecosystems (Yang et al., 2016), affecting energy flow and material conversion in soil environments and participating in many important biochemical reactions (Ling et al., 2014). Bacteria are one of the main components of microorganisms, and their stable community structure and functional diversity play an important role in maintaining soil microecological balance (Jacobsen and Hjelms, 2014). Therefore, studying soil microecosystem have important theoretical and practical significance in tackling challenges associated with continuous cropping (Xia et al., 2018). We speculate that, by applying microbial agents, the abundance, community composition, and functional diversity of rhizosphere microorganisms will be improved. *Bacillus* and *Trichoderma* as antagonists of plant pathogen are widely used to improve plant health and productivity, combining the use of biocontrol agents has potential synergistic effects (Huang et al., 2021). Our previous pot experiment results showed that the combination of AMF, *Bacillus* and *Trichoderma* had the best control effect than the single use, so the mixed bacteria were selected to carry out the study. In this study, a compound microbial agent with excellent promotion and disease prevention ability for ginger previously selected in a previous screening was applied under field conditions with both new planting and continuous cropping modes. The impact of the exogenous compound microbial agent on rhizosphere microorganisms was analyzed. High-throughput sequencing technology was used to explore the response of ginger rhizosphere bacterial communities to the compound microbial agent and the correlation between the changes in bacterial community structure and ginger disease prevention. This study provides a theoretical basis for exploring the field disease prevention mechanism of compound microbial agents on ginger.

## 2. Materials and methods

### 2.1. Experiment materials

The ginger variety used in the field experiment was Shandong Laiwu ginger, and the microbial agents applied were the experimental samples from Guangxi Academy of Agricultural Sciences, including a mix of arbuscular mycorrhizal (AM) agents in the ratio 1:1:1 (*Glomus reticulatum* LCGX-39, *Glomus mosseae* FSGX-1631, and *Glomus versiform*e LCGX-58), *Bacillus velezensis* KC-5, and *Trichoderma viride* BJM-11. There was no mutual inhibition among the strains.

### 2.2. Experiment site

The experiment was conducted at the Wu-Ming Li-Jian Research Station of Guangxi Academy of Agricultural Sciences (23°15′N, 108°02′E), and the tested soil was red soil. Before the experiment, the basic physical and chemical properties of the soil were as follows: available N 91.83 mg/kg, available P 81.86 mg/kg, available K 256.53 mg/kg, organic matter 23.67 g/kg, and pH 6.56.

The experimental area is in a subtropical monsoon climate zone with abundant light, heat, and water resources. The average annual temperature is 21.7°C, the average annual sunshine hours are 1,660, and the average annual rainfall is 1,300 mm.

### 2.3. Experiment design

The field for the continuous cropping experiment was used for planting ginger for two consecutive years, and the field for the new planting experiment was used for planting ginger for the first time.

There were four treatments in the experiment, including new planting plus microbial agent treatment (NT), new planting only control (NK), continuous cropping plus microbial agent treatment (CT), and continuous cropping only control (CK). Each treatment had three replicates, and a randomized complete block design was employed. The soil preparation was conducted on February 13, 2021, and the plastic film was removed on February 28. The ginger was planted on March 7. The AM agent was applied by spreading before planting ginger, with approximately 50 g of the agent per block (the spore count of the agent was approximately 85 spores per gram). The plants were irrigated with *Bacillus velezensis* (at a concentration of 10^8^ CFU/mL) and *Trichoderma viride* (at a concentration of 10^6^ CFU/mL) suspension since a month after planting for three times at April 7, May 7, and June 7, respectively, during the whole growth duration, with a rate of 200 kg/ha each time.

The land with a deep, loose, and fertile loamy and sandy soil layer and good drainage was selected, and the soil was well prepared. After application of base fertilizer, the land was ridged to a height of 60 cm and a width of 4.5 m. A drainage ditch with a width of 60 cm was built between each ridge. Before being planted, the pieces of ginger were first washed with tap water, then soaked in multi-bactericide (diluted at a ratio of 1:1,000) for 30 min before being dried at room temperature for 1–2 days. The disinfected ginger pieces were buried in sterile sand in a greenhouse for sprouting at 15°C–25°C and soil humidity 70%–80%. When the sprouts reached a length of 1 cm, they were transplanted to the field.

**Figure 1:**
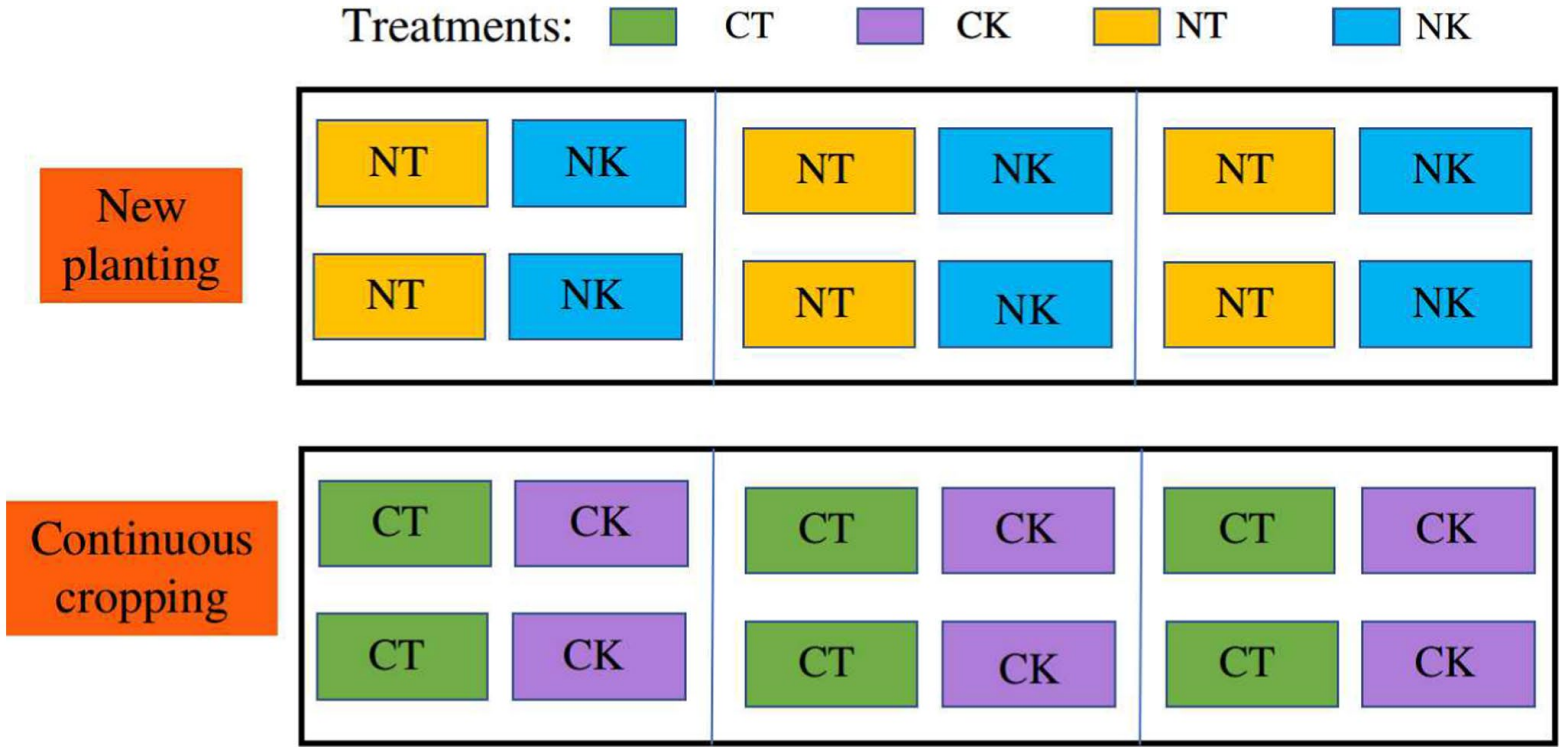
Field experiment design for microbial agent treatments in newly planted and continuous cropping ginger. NT, newly-planted microbial treatment; NK, newly-planted control; CT, continuous cropping microbial treatment; CK, continuous cropping control.

There were four ridges in total. Each ridge was 4.5 m wide and 70 m long divided into four small areas. The ginger is planted in two small beds with a 50 cm aisle in the middle. Each bed is 1.5 m wide, and two rows of ginger had a space of 25 cm × 45 cm between plants. The continuous cropping and new planting experiments were set with two treatments: inoculation and control (Figure 1).

### 2.4. Sample collection and determination

When gingers were harvested on December 10, 2021, the severity index and control efficacy of ginger wilt disease were scored according to NY/T1464.31 “Guidelines for Field Efficacy Trials of Pesticides Part 31: Fungicides for the Control of Ginger Wilt Disease.” The disease severity grading criteria are as follows: grade 0, asymptomatic; grade 1, slight yellowing of 10% of leaves, no obvious symptoms in fleshy stems; grade 3, yellowing of 11% to 30% of leaves, slight curling of leaf edges, water-stained spots appear on fleshy stems; grade 5, yellowing of 31% to 50% of leaves, curling of leaf edges, stunted plants, partial rotting of fleshy stems; grade 7, more than 51% of leaves are withered, plants wilt, and most of the fleshy stems are rot; grade 9, plants die, fleshy stems are rot or only fibrous vascular bundles are left. The disease severity index was calculated as follows: Disease severity index = Σ (Number of plants in each disease grade × Corresponding disease grade)/(Maximum disease grade × Total number of plants) × 100. The control efficacy (%) was calculated as (Disease severity index of control group − Disease severity index of treatment group)/Disease severity index of control group × 100.

The rhizosphere soil of ginger was collected by five-point sampling method at the harvest time in December 2021. The roots with soil were collected and put into a plastic bag, and kept on ice for transportation. In laboratory, the ginger roots were shaken to drop the loose surface soil that is the rhizosphere soil, which was then collected using a sterile brush (Feng et al., 2017). The soil was air-dried and sieved through a 20-mesh sieve, and 300 g was stored for determining the physical and chemical properties of the soil. The dried soil samples were used to measure soil pH using the water extraction method, soil organic matter content using the potassium dichromate heating method, available nitrogen using the alkaline hydrolysis diffusion method, available phosphorus using the molybdenum-antimony colorimetric method, and available potassium using the flame photometry method (Liang et al., 2009).

### 2.5. DNA extraction and illumina sequencing

Genomic DNA was extracted from soil using the Fast DNA TM SPIN Kit (MP Biomedicals, Solon, OH, United States). After extraction, the DNA was checked for quality using 0.8% agarose gel electrophoresis and a NanoDrop 1000 spectrophotometer (Thermo Fisher Scientific, Waltham, MA, United States).

The *Ralstonia solanacearum* in ginger rhizosphere soil of different treatments was determined by real-time fluorescence quantitative PCR. The flic gene of *R. solanacearum* was amplified using a primer pair flicF/flicR (5′-GAA CGC CAA CGG TGC GAA CT-3′/5′-GGC GGC CTT CAG GGA GGT C-3′) (Zhao et al., 2019). The plasmid containing the flic gene of *R. solanacearum* was diluted 10 times to prepare a standard curve of 10^3^–10^9^ copies/μL.

The SYBR^®^ *Premix Ex Taq*^™^ real-time fluorescent PCR kit from TaKaRa company was used to run on the ABI 7500^™^ real-time fluorescent quantitative PCR detector. The amplification reaction system was 20 μL, SYBR® *Premix Ex Taq*^™^ (2 ×) 10 μL, ROX reference dye (50×) 0.4 μL, upstream and downstream primers (10 mmol L^−1^) 0.8 μL, DNA template 2 μL, double distilled water 6.0 μL. Three sample replicates were set in each group, and three replicates were measured. The copy number of flic gene in 1 g dry soil sample was calculated according to the measured Ct value, and the results were presented in logarithmic form l g (copies/g, dry soil).

The V3–V4 region of the bacterial 16S rRNA gene was amplified with PCR using 338F (5′-ACT CCT ACG GGA GGC AGC A-3′) and 806R (5′-GGA CTA CHV GGG TWT CTA AT-3′) primers (Yang et al., 2019). The PCR reaction mixture (25 μL) contained 0.25 μL of DNA polymerase, 5 μL of reaction buffer, 5 μL of high GC buffer, 2 μL of dNTP (10 mM), 2 μL of template DNA, 1 μL of each primer (10 μM), and 8.75 μL of ddH_2_O. The PCR conditions were as follows: 98°C for 30 s; 98°C for 15 s, 50°C for 30 s, 72°C for 30 s, 27 cycles; 72°C for 5 min. The PCR products were then used to prepare sequencing libraries using the TruSeq Nano DNA LT Library Prep Kit and sequenced on an Illumina MiSeq platform. Raw reads were deposited in the NCBI Sequence Read Archive (SRA) database (Accession Number: PRJNA977085).

### 2.6. Sequence data analyses

The Illumina MiSeq raw paired-end sequencing data were in FASTQ format. The quality control was performed by removing sequences with a quality score less than Q20. The quality-filtered sequences were then assembled using FLASH with a minimum overlap of 10 bp and no mismatches. The sequences containing ambiguous bases or chimeras were removed using QIIME2 (Hugo et al., 2020). The filtered sequences were aligned to the 16S rRNA gene sequences in the GreenGenes database using the UCLUST algorithm implemented in QIIME2 with a 97% similarity threshold for operational taxonomic units (OTU) clustering.

### 2.7. Data processing and statistical analyses

To test the effects of treatments on crop yield, soil physical and chemical properties, the least significant difference test was performed at 5% level using SPSS Statistics for Windows (SPSS 19.0) software package. The OTU table with 97% similarity was selected, and the bacterial community composition analysis and Venn analysis were performed using the R language (v.3.3.1) tool, and statistics and plotting were performed. Multivariate statistical methods such as principal coordinates analysis (PCoA) were used for analysis of differences in bacterial communities. The Metastats and LEfSe software was used to screen biomarker features of each group (Segata et al., 2011). To study the relationship between the soil physicochemical indices and samples and microbial communities, redundancy analysis (RDA) was used to correlate the biochemical indices as environmental factors with the sample communities.

## 3. Results

### 3.1. Soil properties and ginger yield

In this study, soil properties, including alkaline nitrogen, available phosphorus, available potassium, organic matter, and pH in the ginger rhizosphere soils with four treatments were measured and the results were shown in Table 1. It can be seen from Table 1 that regardless of the microbial treatment (CT) or control (CK), continuous cropping of ginger significantly reduced the main physicochemical indicators of soil in the newly-planted microbial treatment (NT) compared to the control (NK) (*p* < 0.05). This indicates that continuous ginger cultivation led to more consumption of soil nutrients. There were also significant differences in soil physicochemical properties between the microbial treatment and control. The effective P, available K, and organic matter in NT were higher than those in NK, especially the organic matter, which was 13% higher in NT than in NK (*p* < 0.05). The alkaline nitrogen in CT was higher than that in CK, and the other indicators showed no significant difference. There was no significant difference in soil pH between the newly-planted and continuous cropping treatments.

**Table 1:** Effects of different planting systems on the physicochemical and biological characteristics of the ginger rhizosphere soil.

Treatment	Soil chemical property				
	Available N (mg/kg)	Available P (mg/kg)	Available K (mg/kg)	OM (g/kg)	pH
NT	92 ± 9.97a	56.4 ± 20.92a	688.77 ± 260.24a	24.87 ± 1.91a	6.9 ± 0.44a
NK	92.53 ± 13.71a	48.87 ± 18.72b	585.1 ± 170.85b	21.97 ± 0.68b	6.51 ± 0.18b
CT	68.97 ± 18.19b	28.23 ± 16.23c	153.97 ± 22.15c	17.4 ± 1.66c	6.85 ± 0.14ab
CK	62 ± 1.57c	29.9 ± 6.22c	182.07 ± 24.07c	16.73 ± 1.42c	7.1 ± 0.08a

The values were mean ± standard error, *n* = 3. Different lowercase letters followed the data in the same column indicate significant differences at *p* ≤ 0.05 levels according to the least significant difference (LSD) means comparison test. OM (organic matter). Treatments: NT (newly-planted microbial treatment); NK (newly-planted control); CT (continuous cropping microbial treatment); CK (continuous cropping control).

As indicated in Table 2, the three treatments including NT, NK, and CT significantly reduced the disease index of ginger wilt (*p* < 0.05) compared to CK. Among them, NT had the lowest disease index (5.2) and the highest correctional efficiency (85.87%). NK had a disease index of 8.4 and a correctional efficiency of 77.17%, followed by CT with a disease index of 15.6 and a correctional efficiency of 57.61%. The flic gene abundance of *R. solanacearum* in the rhizosphere soil of NT treatment was 2.51 × 10^6^ copies/g, dry soil, and the flic gene abundance of *R. solanacearum* in the rhizosphere soil of NK treatment was 10.62 × 10^6^ copies/g, dry soil, it is 4.23 times that of NT. The flic gene abundance of *R. solanacearum* in rhizosphere soil of CT treatment was 15.23 × 10^6^ copies/g, dry soil, and the flic gene abundance of *R. solanacearum* in rhizosphere soil of CK treatment was 38.47 × 10^6^ copies/g, dry soil, it is 2.53 times that of CT. The abundance of flic gene in the rhizosphere of NT and CT was significantly lower than that of NK and CK (*p* < 0.05), respectively. Under the same microbial application conditions, the yield in NT was significantly increased by 39% compared to that in CT, and the yield in NK was significantly increased by 56% compared to that in CK.

**Table 2:** Effects of different treatments on the control of ginger wilt disease and yield.

Treatments	Disease index	Flic gene copies of Rs (copies/g)	Control efficacy (%)	Yield (kg/hm^2^)
NT	5.2 ± 0.61d	2.51 × 10^6^d	85.87 ± 1.54a	6,479 ± 86.02a
NK	8.4 ± 0.36c	10.62 × 10^6^c	77.17 ± 0.95b	5,464 ± 36.86b
CT	15.6 ± 0.8b	15.23 × 10^6^b	57.61 ± 1.69c	3,889 ± 104.7c
CK	36.8 ± 0.51a	38.47 × 10^6^a	/	2,357 ± 193.71d

The values were mean ± standard error, *n* = 3. Different lowercase letters followed the data in the same column indicate significant differences at *p* ≤ 0.05 levels according to the least significant difference (LSD) means comparison test. Treatments: NT (newly-planted microbial treatment); NK (newly-planted control); CT (continuous cropping microbial treatment); CK (continuous cropping control).

### 3.2. Taxonomic characterization of rhizosphere microbiota

The V3–V4 region of bacterial 16S rDNA gene was amplified, and the structure and composition of bacterial populations were analyzed by high-throughput sequencing. A total of 1,269,686 sequences with an average length of 416 bp were obtained from 12 soil samples using Illumina MiSeq. After quality control and chimera filtering, 634,843 high-quality sequences were obtained. Finally, 601,196 effective tags were detected in all the samples, accounting for 94.7% of the total quantified sequences. The quality reads of soil samples ranged from 53,462 to 69,760 in the 12 replicates (Supplementary Table S1). These sequences were clustered into operational taxonomic units (OTUs) at a 3% difference level and annotated with species names, resulting in a total of 23,872 OTUs. In the sparse curve analysis, all the curves tended to saturate (Supplementary Figure S1), and effective coverage of almost all the bacterial diversity was found at 97% sequence similarity by rank-abundance curve analysis (Supplementary Figure S2).

### 3.3. Species composition analysis

**Figure 2:**
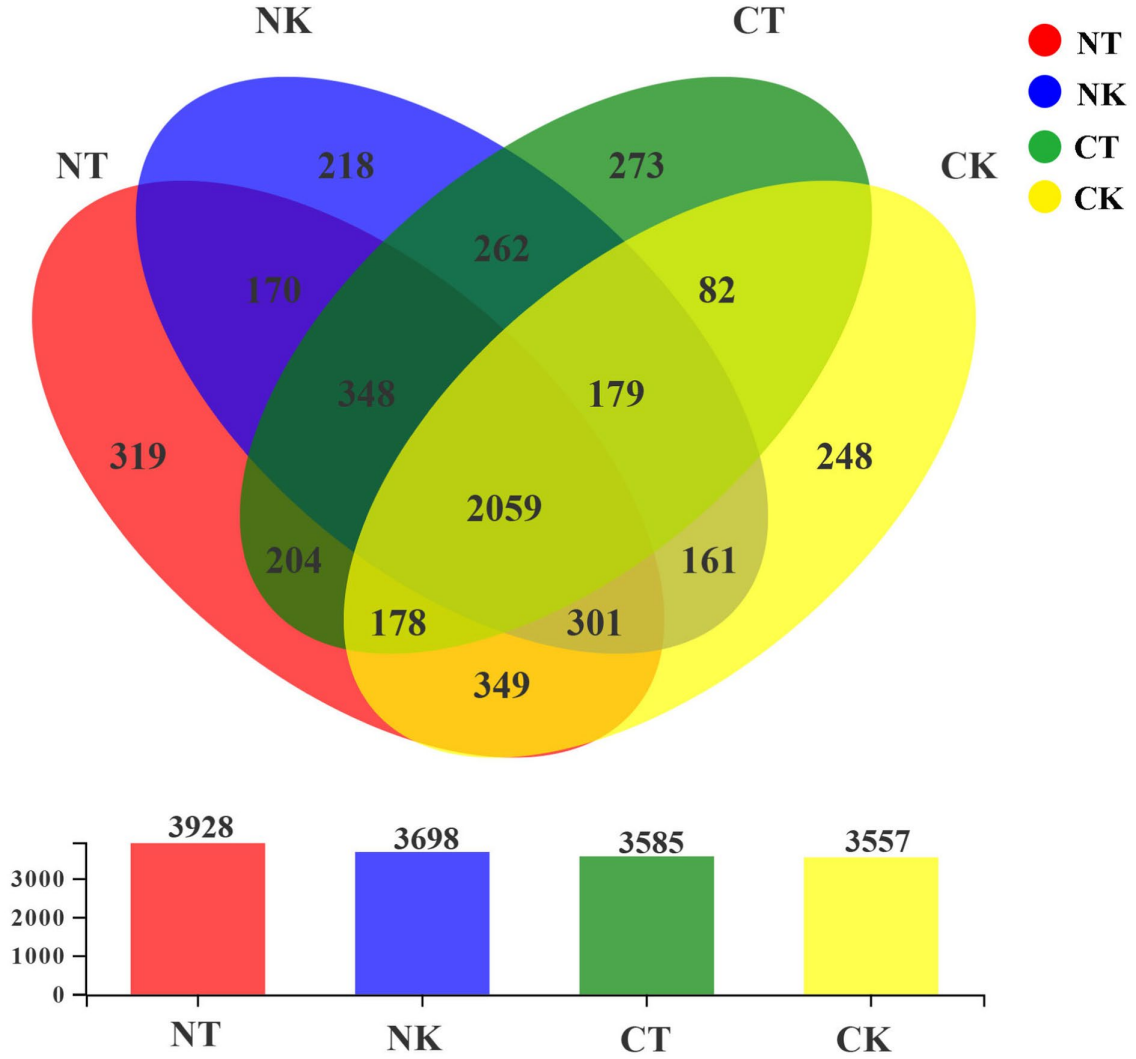
Venn diagram of different treatments (97% sequence similarity). NT, newly-planted microbial treatment; NK, newly-planted control; CT, continuous cropping microbial treatment; CK, continuous cropping control.

At the similarity level of 97%, OTUs were obtained for each treatment, and a Venn diagram was used to visualize the number of the shared and unique OTUs in different treatments. As shown in Figure 2, there were 2,059 shared OTUs among the four treatments, and the numbers of OTUs unique to NT, NK, CT, and CK were 319, 218, 273, and 248, respectively, indicating significant changes in the bacterial colony diversity in different treatments (Figure 2).

### 3.4. Changes in bacterial community composition and diversity

**Figure 3:**
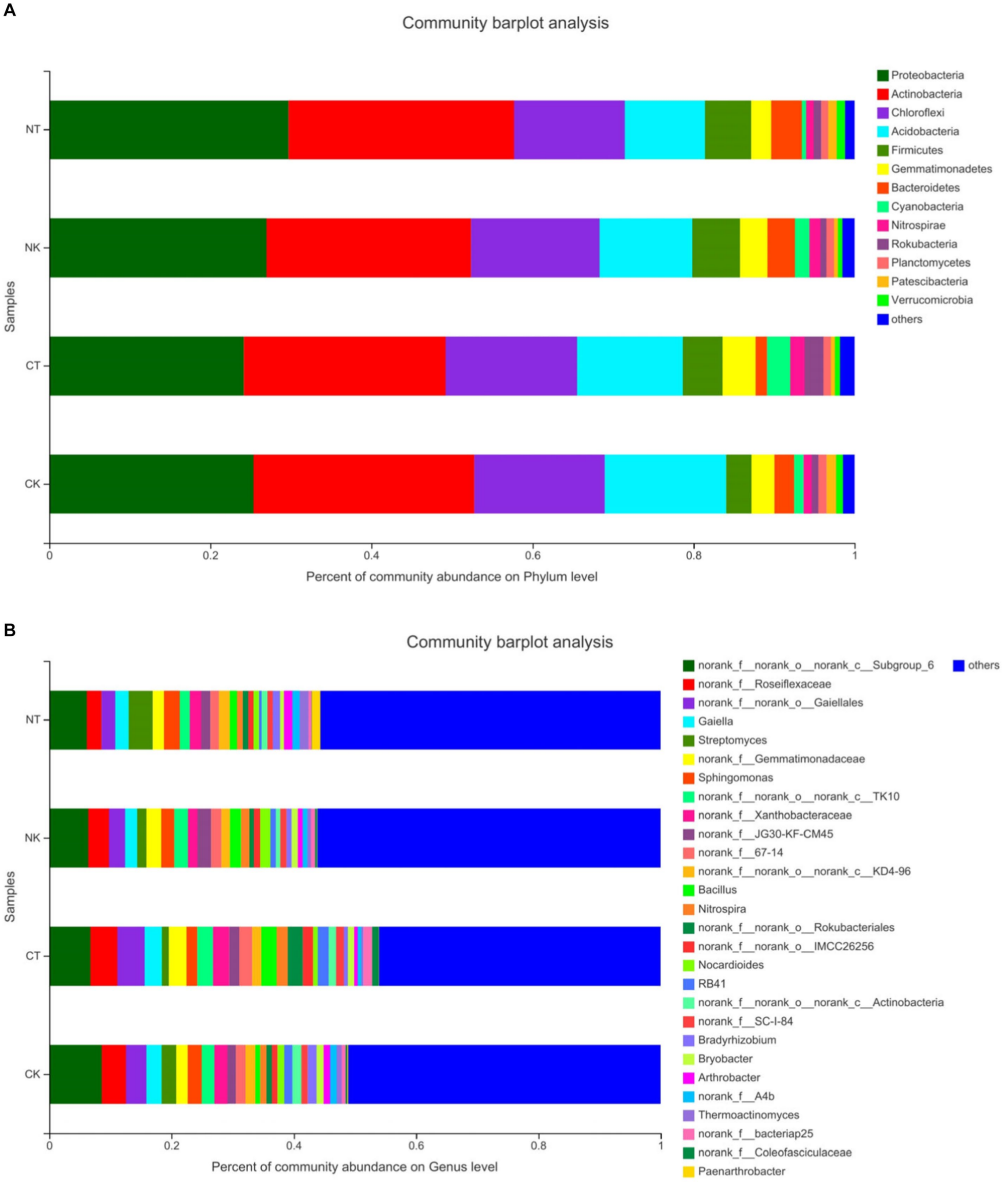
Main community composition of bacteria at phylum level **(A)** and genus level **(B)** under different treatments. NT, newly-planted microbial treatment; NK, newly-planted control; CT, continuous cropping microbial treatment; CK, continuous cropping control.

The OTUs were classified into 32 bacterial phyla and 94 classes. At the phylum level (Figure 3A), the top 13 phyla were *Proteobacteria* (24.16% to 29.71%), *Actinobacteria* (25.05% to 28%), *Chloroflexi* (13.73% to 16.32%), *Acidobacteria* (9.97% to 15.06%), *Firmicutes* (3.14% to 5.92%), *Gemmatimonadetes* (2.47% to 4.07%), *Bacteroidetes* (1.42% to 3.82%), Cyanobacteria (0.51% to 2.89%), *Nitrospirae* (0.93% to 1.79%), *Rokubacteria* (0.74% to 2.34%), Planctomycetes (0.88% to 1.03%), *Patescibacteria* (0.49% to 1.15%), and *Verrucomicrobia* (0.56% to 1.04%). *Proteobacteria* and *Actinobacteria* were the dominant phyla, accounting for 49.21% to 57.71% of the total bacteria, indicating their high abundance. The phylum-level abundance of *Proteobacteria*, *Actinobacteria*, *Firmicutes*, and *Bacteroidetes* showed significant changes between the new planting and continuous cropping treatments. No matter new planting or continuous cropping, the application of microbial agents increased the abundance of *Chloroflexi*, *Acidobacteria*, *Gemmatimonadetes*, *Cyanobacteria*, and *Nitrospirae*. Therefore, the microbial agents significantly altered the relative abundance of dominant bacterial taxa, affecting the composition and structure of the bacterial community.

From Figure 3B, it can be seen that the top 10 genera in terms of abundance were *Roseiflexaceae*, *Gaiellales*, *Gaiella*, *Streptomyces*, *Gemmatimonadaceae*, *Sphingomonas*, *Xanthobacteraceae*, *Bacillus*, *Nitrospira*, and *Rokubacteriales*, followed by *Nocardioides*, *Bradyrhizobium*, *Bryobacter*, and *Arthrobacter*. The abundance of *Roseiflexaceae*, *Gaiellales*, and *Gaiella* in the continuous cropping group (CK and CT) was higher than that in the new planting group (NK and NT), while the abundance of *Streptomyces* in the new planting group (NK and NT) was higher than that in the continuous cropping group (CK and CT). There was little difference in the abundance of bacterial agents and control treatments in the continuous cropping group, but in the new planting group, the abundance NT was significantly higher at 3.97% compared to NK at 1.52%. *Bacillus* is a potential beneficial bacterium that can increase plant disease resistance. In the continuous cropping group, the abundance of *Bacillus* in CT was 2.5%, which is significantly lower in CK at 0.8%. However, in the new planting group, there was little difference in the abundance of *Bacillus* between NT and NK. These data suggest that the microbial agents can significantly change the relative abundance of dominant bacterial genera in the community.

### 3.5. PCoA analysis of OTUs

**Figure 4:**
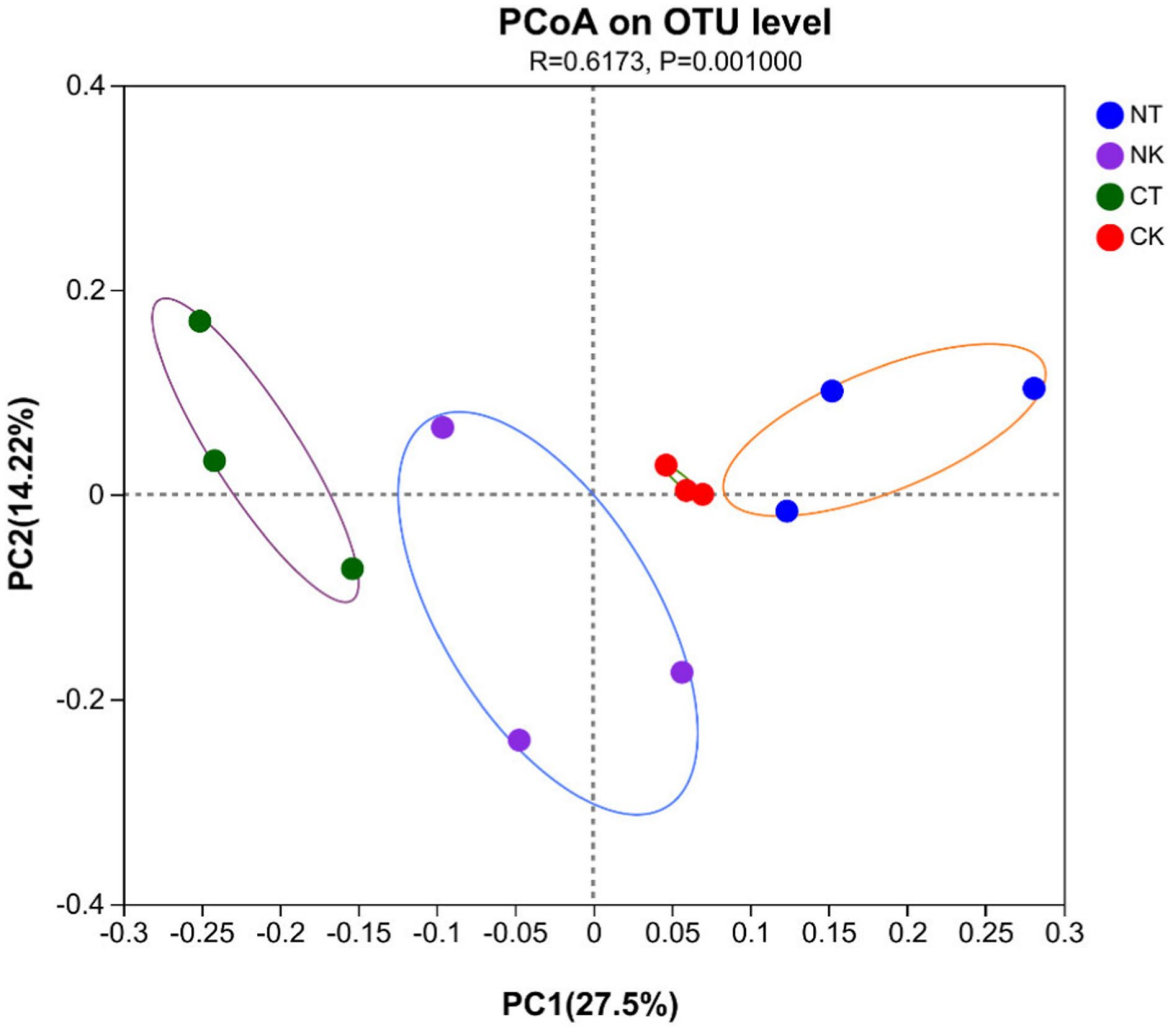
Principal coordinate analysis (PCoA) of bacterial community structure based on the Bray–Curtis distance metric in all soil samples. NT, newly-planted microbial treatment; NK, newly-planted control; CT, continuous cropping microbial treatment; CK, continuous cropping control.

Principal co-ordinates analysis (PCoA) of sequencing showed significantly separate clustering of the soil microbiota community between each pair of control and microbial agent treated groups (*p* = 0.001), with the main principal component (PC) scores: PC1 = 27.5%, PC2 = 14.22%, demonstrating the effects of the microbial agent treatments on the indigenous bacterial community in the rhizosphere soil of field ginger (Figure 4).

### 3.6. Comparative assessment of microbial biomarkers

**Figure 5:**
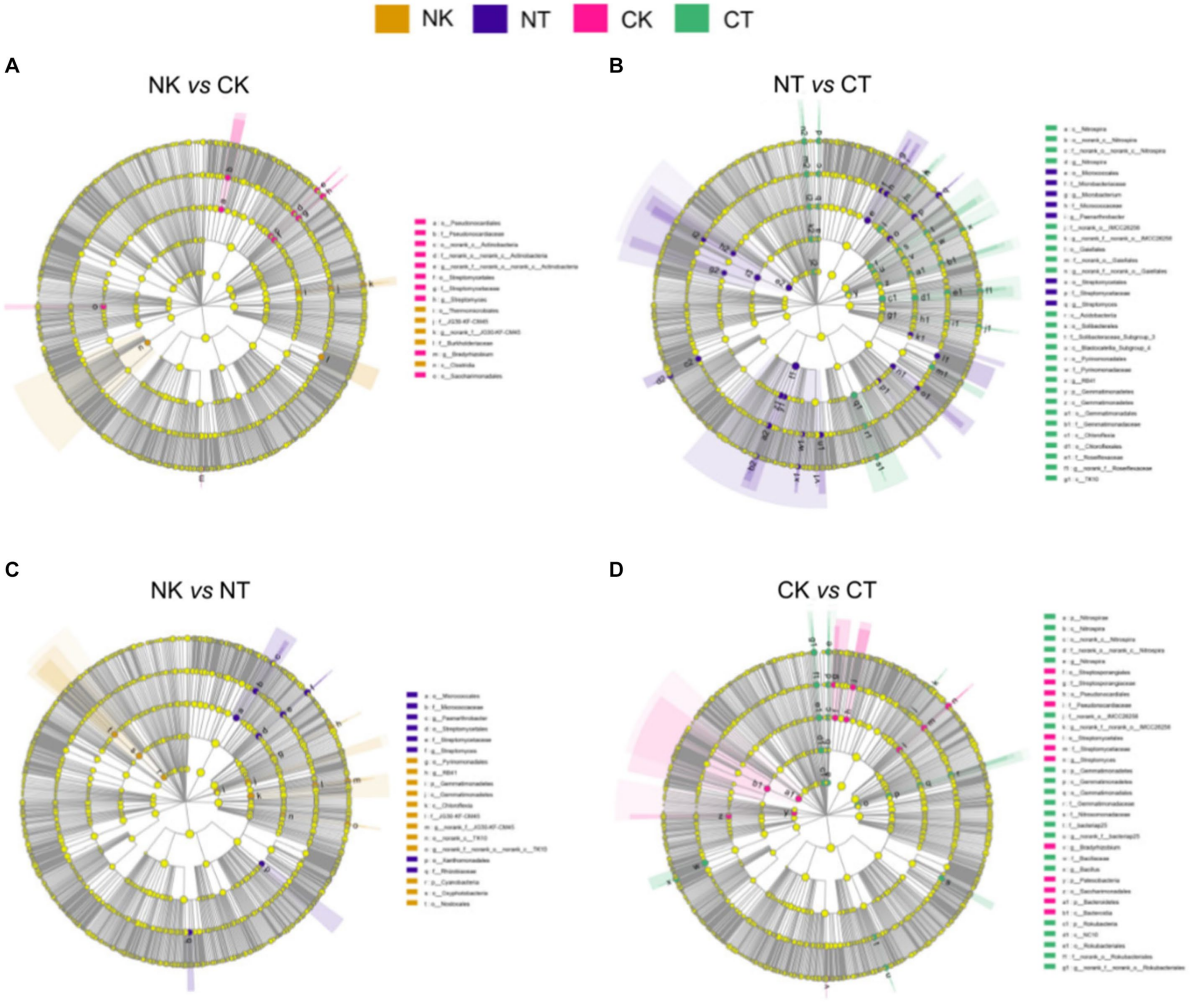
Cladograms plotted from LEfSe comparison analysis indicating the taxonomic representation of statistically and biologically consistent differences of identified biomarkers among different cropping systems. NT, newly-planted microbial treatment; NK, newly-planted control; CT, continuous cropping microbial treatment; CK, continuous cropping control.

LEfSe analysis was conducted with the LDA threshold of 3.5 to identify the microbial biomarkers that differed significantly between the microbial agents. The results showed that *Streptomyces* and *Streptomycetales* were significantly enriched in NT compared to NK, while *Oxyphotobacteria* and *Chloroflexia* were significantly enriched in NK (Figure 5C). In addition, *Rokubacteria* and *Bacillaceae* were enriched in CT, while *Streptomycetaceae* and *Pseudonocardiales* were enriched in CK (Figure 5D). When comparing NT and CT, *Alphaproteobacteria* and *Streptomycetales* were significantly enriched in NT, while *Gaiellales* and *Blastocatellia* were significantly enriched in CT (Figure 5B). Finally, when comparing NK and CK, *Thermomicrobiales* and *Burkholderiaceae* were significantly enriched in NK, while *Streptomycetaceae* and *Streptomycetales* were significantly enriched in CK (Figure 5A). The results suggest that different cropping systems have a significant impact on the composition of microbial communities in the ginger rhizosphere soil.

### 3.7. Environmental factors and microbial association

**Figure 6:**
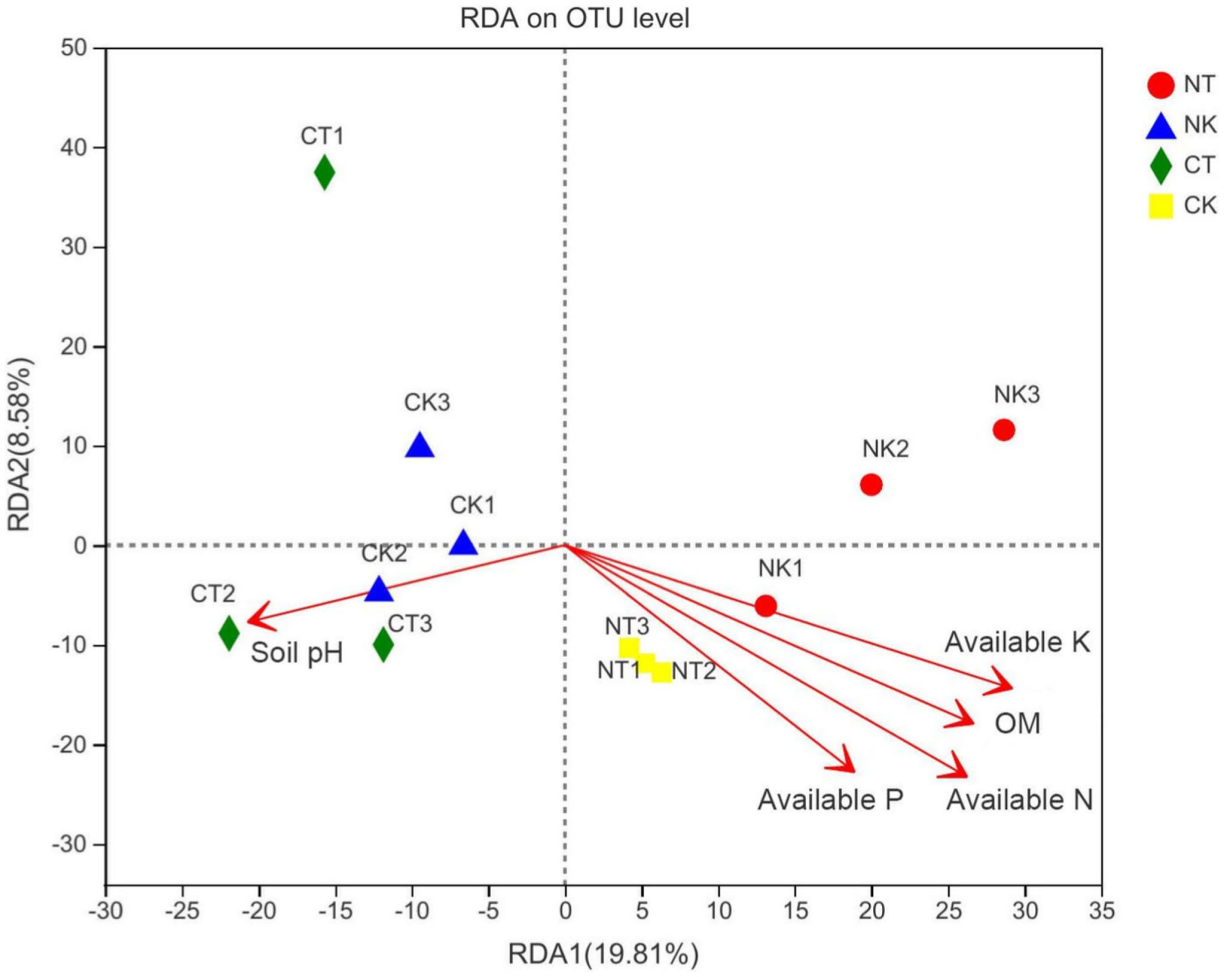
Redundancy analysis (RDA) of soil bacterial community structure associated with soil properties. NT, newly-planted microbial treatment; NK, newly-planted control; CT, continuous cropping microbial treatment; CK, continuous cropping control.

The results of RDA indicated that soil properties, including hydrolyzable nitrogen (*R*^2^ = 0.6913^**^), available phosphorus (*R*^2^ = 0.4981^**^), available potassium (*R*^2^ = 0.5821^**^), and organic matter (*R*^2^ = 0.5715^**^), were the significant predictors of bacterial community composition at the genus level in the ginger rhizosphere soil (Supplementary Table S2). The magnitude of the effects of soil physicochemical properties on the bacterial community composition followed the order of hydrolyzable nitrogen, available potassium, organic matter, and available phosphorus. Available phosphorus was positively correlated with hydrolyzable nitrogen, available potassium, and organic matter, but negatively correlated with pH (Figure 6).

**Figure 7:**
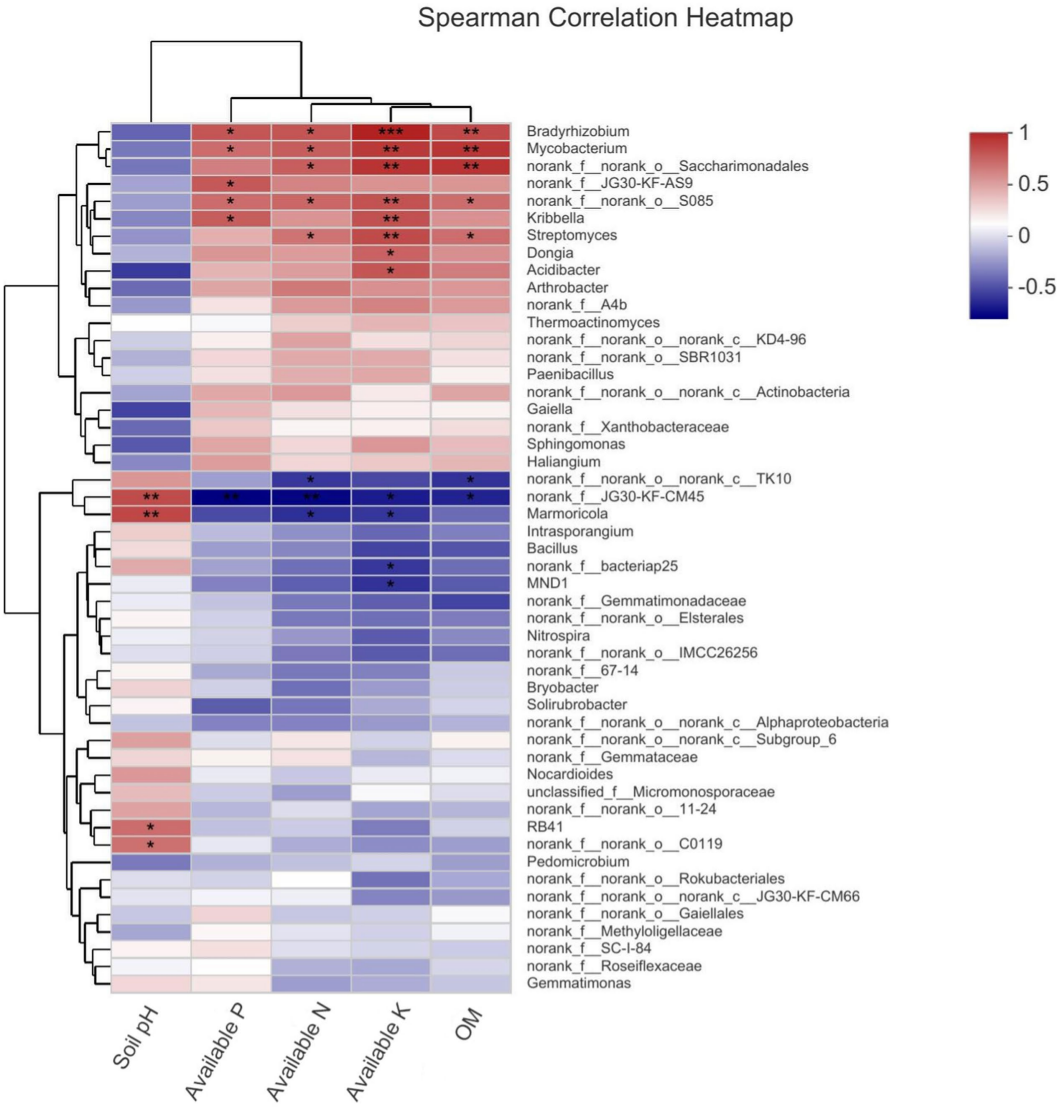
Heatmap correlation between dominant bacteria genara and soil physico-chemical properties.

By calculating the Spearman correlation between the environmental factors and bacterial genera, it was found that the abundance of 12 genera was significantly and positively correlated with the environmental factors, while the abundance of 5 genera was significantly and negatively correlated with the soil physicochemical factors. Specifically, norank_f_JG30-KF-CM45, *Marmoricia*, RB41, and norank_f_norank_o_11–24 were significantly and negatively correlated with pH; *Bradyrhizobium*, *Mycobacterium*, norank_f_JG30-KF-AS9, norank_f_norank_o_S085, and *Kribbella* were significantly and positively correlated with available phosphorus, and norank_f_JG30-KF-CM45 was significantly and negatively correlated with available phosphorus; *Bradyrhizobium*, *Mycobacterium*, norank_f_norank_o_Saccharimonadales, norank_f_norank_o_S085, and *Streptomyces* were significantly and positively correlated with hydrolysable nitrogen, while norank_f_norank_o_norank_c_TK10 was significantly and negatively correlated with hydrolysable nitrogen; *Bradyrhizobium*, *Mycobacterium*, norank_f_norank_o_Saccharimonadales, norank_f_norank_o_S085, *Kribbella*, *Streptomyces*, *Dongia*, and *Acidbacter* were significantly and positively correlated with available potassium, while norank_f_JG30-KF-CM45, *Marmoricia*, norank_f_bacteriap25, and MND1 were significantly and negatively correlated with available potassium; *Bradyrhizobium*, *Mycobacterium*, norank_f_norank_o_*Saccharimonadales*, norank_f_norank_o_S085, and *Streptomyces* were significantly and positively correlated with organic matter, while norank_f_norank_o_norank_c_TK10 and norank_f_JG30-KF-CM45 were significantly and negatively correlated with organic matter (Figure 7).

## 4. Discussion

### 4.1. Effects of microbial agents on rhizosphere soil physicochemical properties and yield of ginger

The results of soil physicochemical properties showed that regardless of the cultivation method or inoculant treatment, the changes in soil pH were not significant, and the impact on the rhizosphere microorganisms was also weak. This is likely due to the short-term effects of continuous cropping, the buffering effect of the soil on pH, and the complexity of the field environment. It has been reported that different crop cultivation methods result in different nutrient utilization and transformation efficiencies in the soil, which in turn lead to different fertility properties (Xun et al., 2015; Liu et al., 2019). The results of this study showed that after 1 year of continuous cropping, the content of available nitrogen, available phosphorus, available potassium, and organic matter in the rhizosphere soil of ginger significantly decreased compared to the newly planted ginger. Therefore, continuous cropping may lead to nutrient loss in soil.

Based on the view of sustainability of agricultural production, one of the most effective measures is to use one or several beneficial bacteria as microbial fertilizers for crop cultivation. These microbial fertilizers not only interact with the crops, but also directly affect the soil physicochemical properties such as soil nutrients and pH, which in turn have feedback effects on the microorganisms and crops (Liu, et al., 2017). In this study, after inoculation of microbial agents in newly planted ginger, the contents of available phosphorus, available potassium, and organic matter in the soil were significantly increased. This is similar to the conclusion of Zhang et al. (2019) that in the first year of planting *Panax notoginseng* (Burk.) Chen, the soil nitrogen content remained basically unchanged, while the phosphorus content increased significantly, and the potassium content decreased significantly. In the present study, however, the microbial agent inoculation treatment in ginger under continuous cropping condition did not cause significant changes in soil physicochemical properties, which may be related to the factors such as soil type, crop species, application cycle, and complexity of the field environment.

### 4.2. Effects of microbial agents on soil bacterial community

Soil microbial community structure and composition are closely related to the soil environment, and planting patterns can cause changes in the soil physical and chemical properties, thus changing the microbial community structure. *Proteobacteria*, *Acidobacteria*, *Bacteriodetes* and *Actinobacteria* are the dominant groups in the soil bacteria in farmland. Microbial agents change the relative abundance of *Proteobacteria*, *Bacteriodetes*, and *Acidobacteria* to some extent (Li et al., 2014). In this study, *Proteobacteria* were the most abundant group in the soil for all the four treatments, with a relative abundance of 24.16% to 29.71%, which is consistent with the results of previous studies (Shen et al., 2015; Zhu et al., 2017). The abundance of bacterial phyla in the soil differed between the continuous cropping and the new planting treatments. Among them, the changes in the relative abundance of *Proteobacteria*, *Actinobacteria*, *Firmicutes* and *Bacteriodetes* were the most obvious, and their abundance in the continuous cropping treatment was significantly lower than that in the new planting treatment. Therefore, different cropping patterns significantly affect the relative abundance of dominant bacterial groups and the composition of bacterial community structure in soil. Based on the results of this study, continuous ginger cropping will reduce the diversity of the rhizosphere soil bacterial community, degrade the structure of the bacterial community, cause an imbalance in the rhizosphere microecology, and thus might trigger the continuous cropping diseases.

The abundance of *R. solanacearum* is an important factor leading to the incidence of ginger (Prameela and Suseela, 2020). Inoculation of microbial agents can significantly reduce the abundance of *R. solanacearum* in the rhizosphere soil of ginger (*p* < 0.05), inhibit the growth of *R. solanacearum* to a certain extent, delay the development of ginger bacterial wilt and reduce its disease index. Inoculation of microbial agents resulted in a decrease in the number of *R. solanacearum* in the soil. It is speculated that there is a complex interaction between microbial agents, *R. solanacearum* and soil bacterial communities. Microbial agents can inhibit the growth of *R. solanacearum* by regulating soil bacterial communities.

Microbial agents (bio-fertilizers, organic fertilizers) can improve soil microbial community structure and control soil-borne diseases by increasing the abundance of beneficial bacteria in the soil (Zhao et al., 2011). Our research found that regardless of whether it was newly planted or continuously cropped, the bacterial inoculant treatment (NT and CT) could increase the abundance of *Chloroflexi*, *Acidobacteria*, *Gemmatimonadetes*, *Cyanobacteria* and *Nitrospirae*. In particular, *Acidobacteria* and *Gemmatimonadetes*, which have strong stress resistance and are beneficial to plants, were found to be significantly increased by 12.2% and 17.1%, respectively, in CT compared to CK in the continuously cropped group. In the newly planted group, the treatment NT increased the abundance of *Acidobacteria* and *Gemmatimonadetes* by 34.4% and 10.7%, respectively, compared to NK. *Acidobacteria* and *Gemmatimonadetes* can improve soil environment, promote ginger growth, and produce antibacterial substances to inhibit the pathogenic bacteria that settle in plant roots, thereby reducing the occurrence of soil-borne diseases (Liu et al., 2021). *Bacillus* is a group of potential beneficial bacteria that can increase plant disease resistance. This study found that the abundance of *Bacillus* sp. in the microbial agent treatment was significantly higher than that in CK in the continuous cropping group. This may be due to the good colonization of *Bacillus velezensis* KC-5 in the soil. *B. velezensis* KC-5 was isolated and screened from the rhizosphere soil of ginger. After applying *B. velezensis* KC-5 to the rhizosphere soil of ginger, the specific root exudates of ginger would promote the growth and reproduction of the bacteria. However, there was little difference in the abundance of *Bacillus* between NT and NK in the new planting. This may be due to the newly planted experimental area shows a milder occurrence of bacteria wilt, the new treatment with *B. velezensis* KC-5 shows colonization in the new area (NT). As for the new control (NK), even though there is no colonization of KC-5, the rhizosphere soil may contain indigenous *B. velezensis*, resulting in no significant difference. However, in the continuous cropping experimental area, the occurrence of bacteria wilt is severe. After applying the microbial agent, the continuous cropping treatment (CT) shows colonization of *B. velezensis* KC-5. On the other hand, the continuous cropping control (CK) not only lacks colonization of KC-5 but also has a reduced abundance of beneficial bacteria in the rhizosphere due to the severe occurrence of bacteria wilt, resulting in significant differences when compared with the continuous cropping control (CK). Nitrospira are the ammonia-oxidizing bacteria that contribute to soil nitrification (Saikia et al., 2018). Their abundance being significantly increased in the microbial agent treatment may have promoted the plant growth. Our research confirmed that the bio-bacterial agents can improve the soil bacterial community structure. Therefore, applying beneficial bacteria to regulate the soil microbial community composition can help prevent ginger wilt disease.

### 4.3. Correlation between soil chemical properties and microbial community under microbial agent treatment

Ginger’s susceptibility to bacteria wilt is influenced by various factors. Wang et al. (2017) demonstrated that temperature and pH are crucial soil factors affecting the pathogenicity of *R. solanacarum*. Other studies (Mahmood and Bashir, 2011) have suggested that severe occurrences of bacteria wilt may be primarily attributed to soil compaction, imbalanced pH levels, and deteriorated physicochemical properties. In this study, the rhizosphere soil of the newly planted experimental area (with less bacteria wilt) showed significantly higher levels of available nitrogen (AN), available phosphorus (AP), and available potassium (AK) compared to the continuous cropping soil (experiencing severe bacteria wilt). This indicates that increasing the content of AN, AP, and AK in the soil plays a vital role in reducing the incidence of bacteria wilt. Many studies have shown the importance of soil physicochemical properties on soil microbial community, and the increase in soil organic matter and available nitrogen content can promote the formation of bacterial diversity (Liu et al., 2014; Li et al., 2018). With the accumulation of soil nutrients, the relative abundance of *Actinobacteria* increases (Zeng et al., 2017). Through correlation analysis, this study found that the abundance of *Bradyrhizobium* and *Mycobacterium* was significantly correlated with the hydrolysable nitrogen, available phosphorus, available potassium, and organic matter in the soil; *Streptomyces* was significantly correlated with the available potassium and organic matter content. More experimental results demonstrated that cropping patterns, soil physicochemical properties, and soil microbial community structure are correlated each other (Ofek-Lalzar et al., 2014; Pang et al., 2017). The above-mentioned research results indicate that different planting patterns and soil chemical properties have inconsistent effects on various soil bacterial communities. As this research focused on analyzing the composition of the bacteria in the rhizosphere soil of mature ginger, further research is needed to investigate the impact of bacterial agents and newly planted ginger on the bacterial community structure at other stages of ginger growth.

Interestingly, high-throughput sequencing results showed that the application of microbial agents did not affect the abundance of *Ralstonia*, so in addition to directly inhibiting pathogens, microbial agents may also reduce the incidence of bacterial wilt through some indirect ways (Tan et al., 2016). Therefore, the specific ways of microbial agents to inhibit pathogens need to be explored in the future.

## 5. Conclusion

High-throughput sequencing results showed that application of microbial inoculant altered the bacterial community structure in ginger rhyzosphere soil. The dominant phyla in the four treatments were *Proteobacteria*, *Actinobacteria*, *Chloroflexi* and *Acidobacteria*, which accounted for 72.91% to 89.09%. The abundance of bacteria at phyla level of *Proteobacteria*, *Actinobacteria*, *Firmicutes*, and *Bacteroidetes* in the new planting treatment was significantly higher than that in the continuous cropping treatment. The microbial agent treatment improved the abundance of the potential probiotics such as *Acidobacteria* and *Gemmatimonadetes* in the soil, which helps optimize the soil microecological balance. The difference in bacterial community structure between the continuous cropping and new planting conditions was greater than that between the microbial and non-microbial inoculant treatments. Correlation analysis showed that the cropping pattern and microbial agent treatments caused significant changes in the physical and chemical properties of the soil, and hydrolyzable N, available P, available K, and organic matter were the significant factors affecting the composition of genus-level bacteria communities in ginger rhizosphere soil. In the future, transcriptome and metabolome analyses associated with specific microbial taxa are required to further explore the potential mechanism, safety, and application strategy of the microbial agents.

## Data availability statement

The datasets presented in this study can be found in online repositories. The names of the repository/repositories and accession number(s) can be found in the article/Supplementary material.

## Author contributions

QW: validation and visualization. QW and JS: investigation and manuscript writing and editing. JS, YK, and JZ: data curation. XQ and SH: project administration. TC and SZ: funding acquisition. All authors contributed to the article and approved the submitted version.

## Funding

This work was supported in part by Guangxi Key Research and Development Project (AB21238002), Guangxi Nature Science Fund (2019GXNSFDA245013), and Guangxi Academy of Agricultural Sciences Project (2023YM96 and 2022JM59).

## Conflict of interest

The authors declare that the research was conducted in the absence of any commercial or financial relationships that could be construed as a potential conflict of interest.

## Publisher’s note

All claims expressed in this article are solely those of the authors and do not necessarily represent those of their affiliated organizations, or those of the publisher, the editors and the reviewers. Any product that may be evaluated in this article, or claim that may be made by its manufacturer, is not guaranteed or endorsed by the publisher.

## Supplementary material

The Supplementary material for this article can be found online at: https://www.frontiersin.org/articles/10.3389/fmicb.2023.1203796/full#supplementary-material
