# Supplementary material for: Effects of microbial agent application on the bacterial community in ginger rhizosphere soil under different planting years
Source: Front Microbiol. 2023 Sep 7;14:1203796. doi: 10.3389/fmicb.2023.1203796 (PMC10516606; doi:10.3389/fmicb.2023.1203796)
Supplement: Supplementary file 1 [file Data_Sheet_1.doc]

Supplementary Table S1 Description of all identified sequences affiliated with soil samples with 16S rDNA Illumina HiSeq analysis

| Sample\Info | Seq_num | Base_num | Mean_length | Min_length | Max_length |
| --- | --- | --- | --- | --- | --- |
| NT1 | 58814 | 24416979 | 415.1558983 | 203 | 476 |
| NT2 | 59267 | 24644777 | 415.8262946 | 203 | 476 |
| NT3 | 31608 | 13167969 | 416.6024108 | 230 | 465 |
| NK1 | 63521 | 26419498 | 415.9175391 | 210 | 507 |
| NK2 | 63191 | 26286644 | 415.9871501 | 203 | 493 |
| NK3 | 41608 | 17290000 | 415.5450875 | 216 | 497 |
| CT1 | 60077 | 25057921 | 417.0967425 | 211 | 477 |
| CT2 | 50786 | 21155087 | 416.5535187 | 248 | 477 |
| CT3 | 54047 | 22518130 | 416.6397765 | 224 | 476 |
| CK1 | 54828 | 22754695 | 415.0196068 | 238 | 510 |
| CK2 | 45758 | 19039094 | 416.0823025 | 235 | 470 |
| CK3 | 51338 | 21392228 | 416.693833 | 203 | 498 |

**
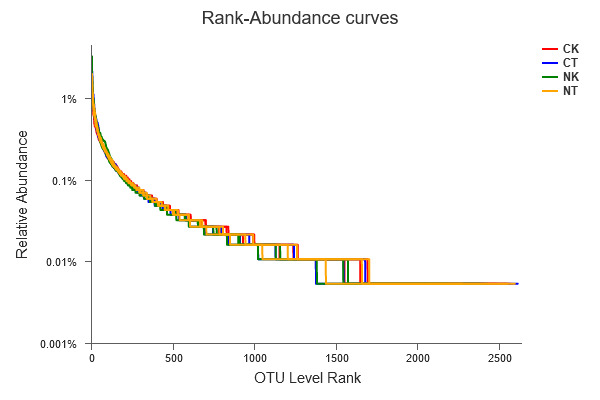
**

FIGURE S1 Rank Abundance curve of different treatments

**
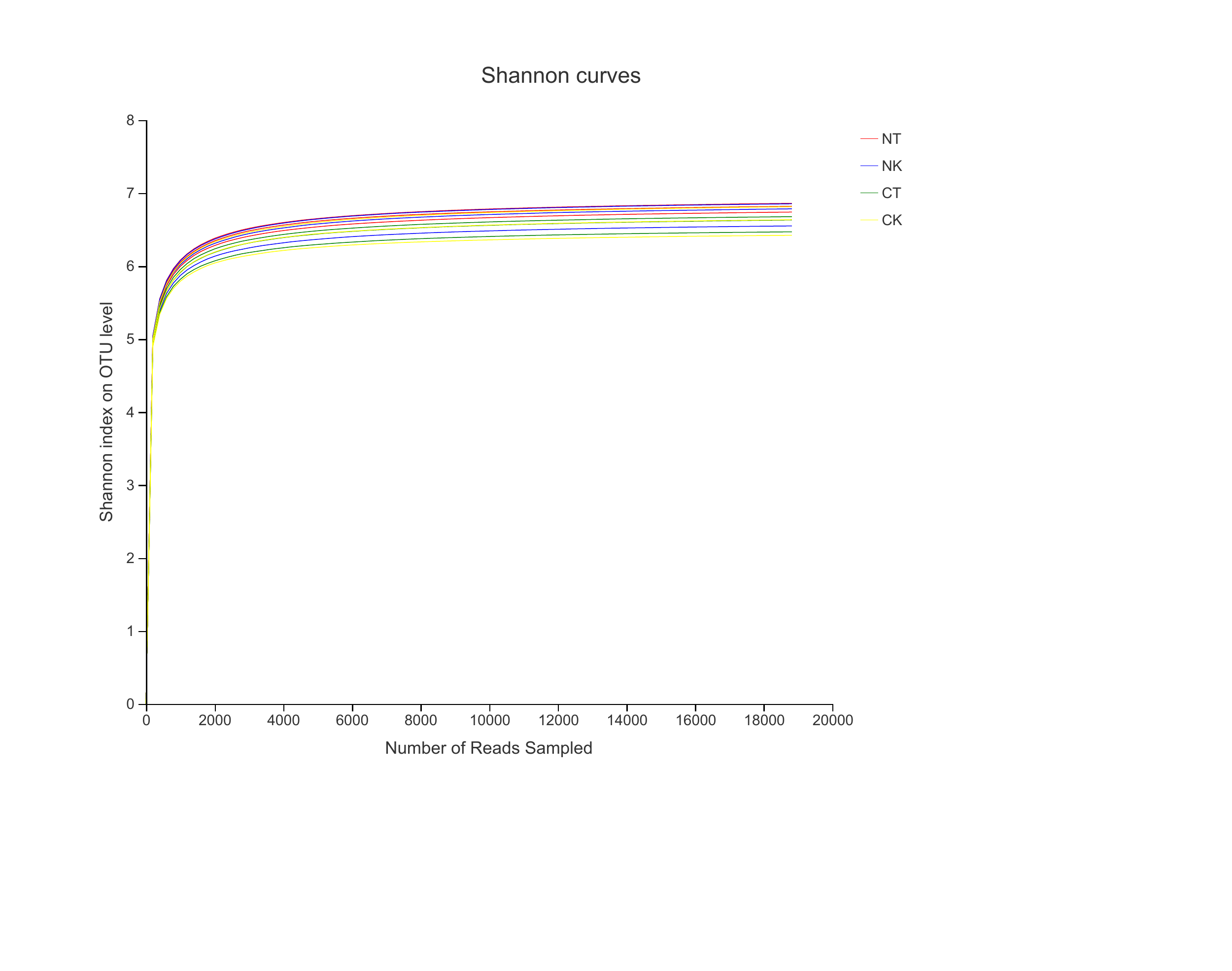
**

FIGURE S2 Dilution curve


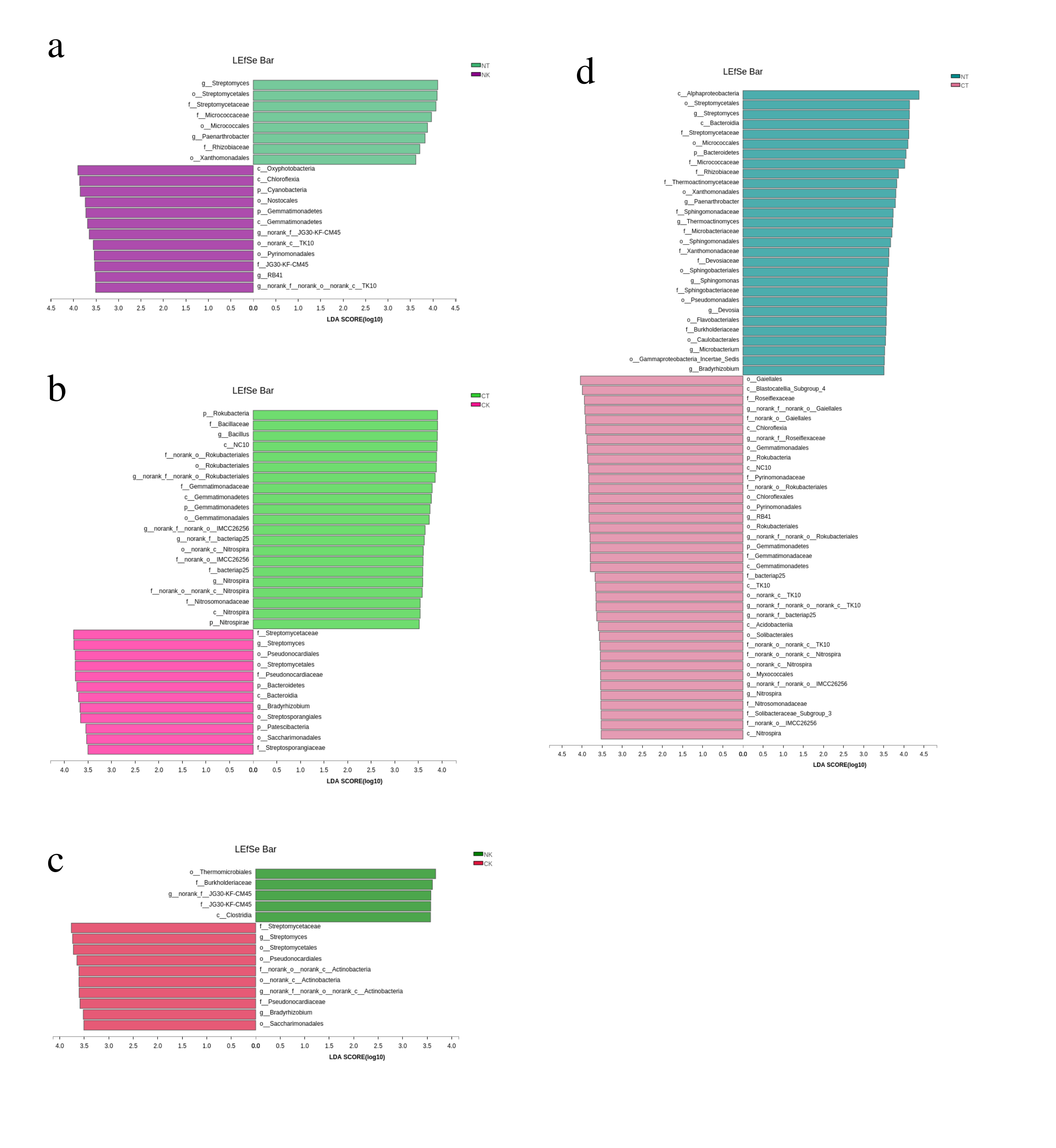


FIGURE S3 The enrichment analysis of bacterial taxa in all the groups at the genus level with a linear discriminant analysis (LDA=3.5)(a. NT&NK; b. CT&CK; c. NK;CK; d. NT&CT)

Supplementary Table S2 RDA related data sheet

|  | RDA1 | RDA2 | r^2^ | *P*_values |
| --- | --- | --- | --- | --- |
| Available N | 0.707 | - 0.7072 | 0.6913 | **0.003** |
| Available P | 0.5802 | - 0.8145 | 0.4981 | **0.039** |
| Available K | 0.8873 | - 0.4612 | 0.5821 | **0.018** |
| OM | 0.8052 | - 0.593 | 0.5715 | **0.022** |
| Soil pH | -0.906 | - 0.4232 | 0.2819 | 0.24 |
